# An Assessment of the Bioactivity of Coffee Silverskin Melanoidins

**DOI:** 10.3390/foods8020068

**Published:** 2019-02-12

**Authors:** Silvia Tores de la Cruz, Amaia Iriondo-DeHond, Teresa Herrera, Yolanda Lopez-Tofiño, Carlos Galvez-Robleño, Marin Prodanov, Francisco Velazquez-Escobar, Raquel Abalo, Maria Dolores del Castillo

**Affiliations:** 1 Instituto de Investigación en Ciencias de la Alimentación (CIAL) (CSIC-UAM), 28049 Madrid, Spain; toresdelacruzsilvia@gmail.com (S.T.d.l.C.); amaia.iriondo@csic.es (A.I.-D.); teresa.herrera@csic.es (T.H.); marin.prodanov@uam.es (M.P.); 2 Departamento de Ciencias Básicas de la Salud, Facultad de Ciencias de la Salud, Universidad Rey Juan Carlos (URJC), Alcorcón, 28922 Madrid, Spain; yolanda.lopez@urjc.es (Y.L.-T.); carlos.galvezrobleno@gmail.com (C.G.-R.); raquel.abalo@urjc.es (R.A.); 3 Technische Universität Berlin, 135/PC14 Max Volmer Laboratorium für biophysikalische Chemie, 1023 Berlin, Germany; velazquez.escobar@chem.tu-berlin.de

**Keywords:** antioxidant, coffee byproduct, dietary fiber, gastrointestinal motility, melanoidins, Maillard reaction, silverskin

## Abstract

Melanoidins present in coffee silverskin, the only by-product of the roasting process, are formed via the Maillard reaction. The exact structure, biological properties, and mechanism of action of coffee silverskin melanoidins, remain unknown. This research work aimed to contribute to this novel knowledge. To achieve this goal, melanoidins were obtained from an aqueous extract of Arabica coffee silverskin (WO2013004873A1) and was isolated through ultrafiltration (>10 kDa). The isolation protocol was optimized and the chemical composition of the high molecular weight fraction (>10 kDa) was evaluated, by analyzing the content of protein, caffeine, chlorogenic acid, and the total dietary fiber. In addition, the structural analysis was performed by infrared spectroscopy. Antioxidant properties were studied in vitro and the fiber effect was studied in vivo, in healthy male Wistar rats. Melanoidins were administered to animals in the drinking water at a dose of 1 g/kg. At the fourth week of treatment, gastrointestinal motility was evaluated through non-invasive radiographic means. In conclusion, the isolation process was effective in obtaining a high molecular weight fraction, composed mainly of dietary fiber, including melanoidins, with in vitro antioxidant capacity and in vivo dietary fiber effects.

## 1. Introduction

Melanoidins are widely distributed in foods and are part of our daily diet. These compounds are found in coffee, bakery products, boiled potatoes, cocoa, toasted barley, beer, caramel, and sweet wine. Coffee and bakery products are the most important sources of melanoidins. It is estimated that the daily intake of melanoidins from these sources is approximately between 1.5 and 6 g for the average consumer [1]. Melanoidins are high molecular weight brown polymeric compounds generated during the last stage of the Maillard reaction [1]. Melanoidins are responsible for color, taste, and texture of foods submitted to high temperatures [2]. There are two main types of melanoidin structures present in foods—melanoidins of polysaccharide type, such as those described in coffee brew; and protein-based melanoidins, predominant in bakery products [1]. Extraction of melanoidins can be carried out by different techniques [3]. Dialysis and diafiltration with membranes from the ultrafiltration scale are the most frequently used procedures for elimination of low molecular weight compounds and recovery of an enriched fraction of high molecular weight compounds, such as polysaccharides, proteins, and melanoidins [4]. The most used technique for the isolation of coffee melanoidins is ultrafiltration, with 10 kDa molecular weight cut-off membranes. Ultrafiltration presents various advantages, such as treatment of huge volume extracts for short time, no phase change, no use of solvents, complete physical separation, and easy industrial scaling. 

Coffee silverskin may be an important source of melanoidins. Coffee silverskin is the tegument of the outer layer of the coffee bean, representing approximately 4.2% (*w/w*) of the coffee cherry and is the only by-product produced during coffee roasting [5]. The roasting of four tons of coffee produces about 30 kg of coffee silverskin [6]. During the roasting process, melanoidins are transformed into a more complex structure, where low molecular weight compounds, such as chlorogenic acids, bind non-covalently to the initial skeleton, constituted by carbohydrates, dietary fiber, polyphenols, and proteins [1]. The total dietary fiber in aqueous coffee silverskin extract (CSE) has been reported to be about 36% [7].

Coffee melanoidins possess various biological properties such as being anticarcinogenic, anticariogenic, antiglycative, antihypertensive, anti-inflammatory, antimicrobial, antioxidant, and prebiotic [1,8,9]. Melanoidins from coffee have been described as “Maillardized dietary fiber” [10]. Coffee silverskin has been proposed as a sustainable natural source of prebiotics, antioxidants, and dietary fiber [11]. Borrelli et al. [12] described for the first time that coffee silverskin supports the growth of bifidobacteria, in vitro, and subsequently Jiménez-Zamora et al. have evaluated the prebiotic properties of different coffee byproducts [13]. Coffee silverskin significantly increased the amount of healthy bacteria, such as *Lactobacillus* spp. and *Bifidobacterium* spp., without affecting the level of *Bacteroides* spp. and *Clostridium* spp. The authors concluded that coffee silverskin could be a suitable ingredient in the formulation of foods with prebiotic characteristics. The antioxidant capacity of coffee silverskin extract is due to the melanoidins generated during the roasting process [14], and also to the presence of chlorogenic acid (CGA) [15]. Determining the contribution of each of these compounds to the overall antioxidant capacity of the extract is of great interest. Additionally, the dietary fiber effect of melanoidins from coffee silverskin has not been investigated yet. Therefore, the aim of the present study was to isolate and perform a structural and functional characterization of melanoidins obtained from coffee silverskin, and to analyze the antioxidant properties in vitro and the dietary fiber effect in vivo.

## 2. Materials and Methods

### 2.1. Raw Material

Coffee silverskin Arabica species from Colombia was kindly provided by Supracafé S.A. (Móstoles, Spain). Coffee silverskin was generated during the roasting process of green coffee beans in a PROBAT roaster (Emmerich am Rhein, Germany), for 13 min, at 220 °C. 

### 2.2. Preparation of Coffee Silverskin Extract

The coffee silverskin extract (CSE) was prepared according to the procedure described in the patent WO2013004873A1 [16]. Briefly, a mixture of 50 g/L of coffee silverskin, in water, was stirred for 10 min at 100 °C, then the suspension was screened by 250 µm and was freeze-dried. The extraction yield of CSE was 10.09%, which corresponded to that previously described [5].

### 2.3. Recovery of Melanoidin-Rich Macromolecular Fraction

A simple, easy to scale up, cost-friendly and sustainable process, avoiding the use of potential harmful solvents for human health, was performed to obtain water soluble melanoidins. Molecules with this physicochemical property are of great interest because of their physiological effect as a soluble dietary fiber. In addition, water solubility facilitates their administration in drinkable water, for the animal model, avoiding stress caused by the use of gavage, in the gastrointestinal tract.

The filtrate obtained after water extraction of coffee silverskin was clarified by dead-end filtration, using cellulose pads (SA-590) from Filtrox Southern Europe S.L. (Besós, Spain) and vacuum as a driving force. The melanoidin fraction (120.32 g) was obtained by aqueous extraction from 3.76 kg of coffee silverskin in 75.2 L of hot water, corresponding to a concentration of 50 g CS/L. 

Melanoidins (MEL) were recovered as the macromolecular fractions of coffee silverskin, through ultrafiltration, using a 10 kDa nominal molecular weight cut-off membrane from Millipore (Merck, Darmstadt, Germany) (model, Prep-scale 6; material, regenerated cellulose; design, spiral-wound; filtration surface, 0.54 m^2^). The membrane was integrated in a tangential (cross) flow, pressure-driven membrane filtration unit, consisting of a 5 L feed vessel, variable flow peristaltic pump (1–13 L/min), membrane holder with integrated inlet, and outlet pressure gauges (1–5 bar). Ultrafiltration was carried out in a continuous concentration mode, at a constant transmembrane pressure of 0.8 bar, and a temperature of 22 °C, until 1.5 L of the concentrate were achieved. Recovery of the macromolecular (colloidal) fraction was carried out by diafiltration of the final concentrate, with the same volume of demineralized water (batch mode), until 0.1% of the total dissolved substances (TDS) (°Brix) were registered in the permeate flow. This effect was achieved within six cycles of dilution/concentration, equivalent to 9 L of water per 1.5 L of concentrate. The purified fraction was freeze-dried and stored at room temperature in a dark place, until analyses.

After filtration, the membrane was cleaned by 0.1 N NaOH solution, according to producer’s requirements.

Both ultrafiltration and diafiltration stages were monitored by the spectrophotometric measurement of caffeine (CAF) at 273 nm and melanoidins at 405 nm (BioTek Instruments, Winooski, VT, USA), and measurement of pH and turbidity.

### 2.4. Microbiological Quality

The melanoidin beverage (4 g/L) prepared form the purified melanoidin-rich macromolecular fraction of coffee silverskin was microbiologically analyzed to evaluate the safety of its use as a food ingredient. A count of the (i) total aerobic microorganisms, (ii) aerobic microorganisms forming endospores, and (iii) molds and yeasts, was carried out. All assays were performed in sterile conditions, with a previous solubilization of the melanoidin fraction (10 g) in buffered peptone water (BPW) (90 mL), by using a stomacher (230 rpm, 1 min). Different conditions were set for each analysis: (i) the pour plate method, plate count agar (PCA) medium, incubation at 30 °C, for 72 h; (ii) the pour plate method, brain heart infusion (BHI) agar medium, pre-incubation at 80 °C, for 10 min and incubation at 37 °C, for 48 h; and (iii) the spread method, sabouraud dextrose agar (SDA) medium with chloramphenicol and incubation at 25 °C, for 120 h. Results were expressed as colony forming units (CFU)/g. 

### 2.5. Spectral Analyses

#### 2.5.1. Ultraviolet-Visible (UV-VIS) Absorption Spectroscopy

UV-VIS spectrum of the caramel reference substance, CSE, MEL, CGA (0.06 mg/mL), and CAF (0.03 mg/mL) were acquired, using a microplate reader (BioTek Epoch 2 Microplate Spectrophotometer, Winooski, VT, USA). Sulphite ammonia caramel (E-150d) was used as a melanoidin standard. Caramel is formed during Maillard-type reactions where carbonyl compounds react with amino groups or ammonia [17]. Therefore, it was used as a melanoidin control. Analytical determination was carried out in triplicates. Absorption spectra were recorded between 240 and 720 nm, at room temperature. Monitorization of the isolation process was carried out by the analysis of the UV-VIS absorption spectra.

#### 2.5.2. Infrared Spectroscopy (IR)

The infrared spectra of the caramel standard, CSE, and MEL were recorded in a Tensor27 FT-spectrometer (Bruker, Billerica, MA, USA), equipped with a diamond-attenuated total reflection (ATR) cell (Durascope, Smiths Detection, Danbury, CT, USA). A background reference was measured (400 scans) previous to sample infrared spectrum recording (200 scans). After each experiment, the diamond crystal was cleaned with distilled water (Millipore quality) and ethanol (chromatographic grade). The infrared spectra were analyzed using the OPUS software (Bruker). The corrected spectra of the baseline were plotted, using the OriginLab software (V10.X, OriginLab Corporation, Northampton, MA, USA). Characteristic infrared red bands were observed within a low window (750–1800 cm^−1^) and high frequency (2700–3400 cm^−1^).

### 2.6. Chemical Analyses

#### 2.6.1. Caffeine and Chlorogenic Acid 

The content of CAF and CGA was determined using capillary zone electrophoresis (CZE), as described by del Castillo et al. [18], in an attempt to identify the bioactive compounds present in samples. Determinations were carried out by an Agilent G1600 A (Santa Clara, CA, USA) capillary electrophoresis instrument equipped with the ChemStation software and a diode array detector (DAD). The capillary was 48.5 cm long (40 cm to the detector) with an internal diameter of 50 µm and a ×3 bubble cell. Other conditions of analysis were as follows: 20 mM borate buffer at pH 9.3; a voltage of 20 kV; temperature of analysis set at 25 °C; injection administered at 50 mbar for 5 s; and an electroosmotic flow (EOF) marker of acetone. Electrophoregrams (e-grams) were monitored at 280 nm, and spectra were collected from 190 to 600 nm. The capillary was conditioned after each sample was run by flushing it with 0.1 M NaOH, for 3 min, and with a buffer for another 3 min. CAF and CGA were used as standards for identification and quantification. All analyses were performed in triplicates; results are expressed as % CAF or CGA (*w/w*).

#### 2.6.2. Melanoidins

The content of melanoidins was determined by CZE, as previously described. Caramel (E-150d) was used as reference. The analyses were performed in triplicates; results are expressed as % (*w/w*) of the caramel equivalents.

#### 2.6.3. Soluble Proteins

A Bio-Rad Protein Assay, catalogue number 500-006 (Bio-Rad Laboratories, SIG 093094), based on the Bradford method, was used in the micro-method format to determine protein concentration. It was performed according to the manufacturer’s instructions. Briefly, a solution of Bradford reagent (1:4, reagent:milli-Q water) was prepared and filtered using a Whatman 4 filter. Ten µL of sample and 200 µL of Bradford solution were placed in a multi-well microplate. Samples were incubated for 5 min at room temperature, and the absorbance was measured at 595 nm. Sample blank and reagent blank were also analyzed. A calibration curve was constructed using BSA (0.05–0.5 mg/mL). All measurements were performed in triplicates. Results are expressed as % (*w/w*).

#### 2.6.4. Dietary Fiber

Insoluble (IDF), soluble (SDF), and total (TDF) dietary fiber content was determined, using the Total Dietary Fiber Assay Kit (Megazyme International Ireland, Ireland) according to the manufacturer’s instructions, and based on the enzymatic–gravimetric method. All measurements were performed in triplicates. Results are expressed as a percentage (%). 

### 2.7. Bioactivity Evaluation

#### 2.7.1. In Vitro

##### 2,2′-Azinobis-(3-Ethylbenzothiazoline 6-Sulfonic acid) (ABTS) Assay

The trapping capacity of cationic free radicals was evaluated using the method of radical ABTS^•+^ bleaching, described by Re et al. (1999) and modified by Oki et al. (2006), for its use in a microplate [19,20]. Aqueous solutions of Trolox (0.025–0.2 mM) and CGA (0.025–0.2 mM) were used for calibration. Absorbance was measured at 734 nm, using a UV-Visible Spectrophotometer (BioTek Instruments, USA). All measurements were performed in triplicates; results are expressed as µmol Trolox equivalents (TE)/mg sample.

##### Oxygen Radical Absorbance Capacity (ORAC) Assay

The ORAC assay was applied, according to the method described by Ou et al. [21], and as modified by Dávalos et al. [22]. The procedure was carried out using an automated plate reader (BMG LABTECH, Germany), equipped with a fluorescence detector set at excitation and emission wavelengths of 485 nm and 530 nm, respectively. Readings were taken every minute, for a duration of 90 min, at 37 °C. All measurements were performed in triplicates; results are expressed as µmol TE/mg sample.

##### Intracellular Reactive Cxygen Species (ROS) 

Normal rat small intestine epithelial cells (IEC-6) were kindly provided by the Bioanalytical Techniques Unit (BAT) of the Instituto de Investigación en Ciencias de la Alimentación (CIAL) (Madrid, Spain). Cells were cultured as a monolayer in Dulbecco’s Modified Eagle Medium (DMEM), supplemented with 10% (*v/v*) heat inactivated fetal calf serum (FBS), 50 U/mL penicillin, 50 µg/mL streptomycin, and 1% (*v/v*) l-glutamine, at 37 °C, and in 5% CO_2_ in a humidified incubator (BINDER CB series 2010, Tuttlingen, Germany).

Prior to the study of basal intracellular ROS, the effect of different concentrations of CSE and melanoidins on cell viability was measured by the MTT assay [23], in order to select non-cytotoxic doses. IEC-6 cells were treated with samples at 0.004, 0.04, 0.4, and 4 mg/mL. DMSO (50%) was used as a death control.

The determination of basal intracellular ROS was performed, following the same procedure used by Iriondo-DeHond et al. [5]. Tert-butyl hydroperoxide (tBOOH) 1 mM was used as a positive oxidation control and vitamin C (10 µg/mL) was used as an antioxidant reference compound. Induced intracellular ROS were also measured, when combining samples, and tBOOH 1 mM. Then, an MTT assay was performed to normalize the data by the number of cells per well. Experiments were carried out in triplicates.

#### 2.7.2. In Vivo

##### Animals and Experimental Design 

Male Wistar rats (150–200 g) were obtained from the Veterinary Unit at Universidad Rey Juan Carlos (URJC) (*n* = 14). Animals were group-housed (2–4 rats/cage) in standard transparent cages (60 cm × 40 cm × 20 cm), under environmentally controlled conditions (temperature = 20 °C; humidity = 60%), with a 12-hour light/12-hour dark cycle. Animals had free access to standard laboratory rat chow (Harlan Laboratories Inc., Barcelona, Spain) and sterile tap water. All experimental procedures were approved by the Ethics Committee of URJC and were carried out in accordance with the EU Directive for the protection of animals used for scientific purposes (2010/63/EU) and the Spanish regulations (RD/53/2013).

Animals were divided in two groups, the control group (*n* = 8) and the melanoidins group (*n* = 6). Although the MEL beverage is within the established limits for molds and aerobes, when compared to tea, it was sterilized in an autoclave, at 121 °C, for 20 minutes, and was stored at 4 °C. A fresh MEL bottle was prepared daily to administer to the animals. MEL were administered in drinking water at 4 g/L, corresponding to a dose of 1 g/kg of body weight [24]. A pilot study validated the oral route for administration of melanoidins in the drinking water. Exposure to normal water or water with melanoidins was performed during four consecutive weeks. Throughout this time, general parameters were regularly evaluated (body weight, water and food intake, and appearance of animals). In the last week, specific parameters of gastrointestinal and colonic motility were also evaluated.

To assess possible dehydration, the dorsal skin fold was evaluated [25]. In addition, the appearance of the perianal area as well as the fecal pellets were evaluated [26]. Each parameter was evaluated separately. This assessment was made every day.

##### Radiographic Study of Gastrointestinal Motility

The analysis of gastrointestinal motility was carried out at the end of the fourth week of the study, through radiographic, non-invasive, in vivo methods, routinely used by the Pharmacology Laboratory of the URJC [27,28]. Radiographs were taken 0, 1, 2, 4, 6, 8 h (T0–T8) after administration of the barium contrast (Barigraf^®^ AD; Juste SAQF, Madrid, Spain) at 3 mL/rat (Barigraf was suspended in distilled water at 2 g/mL). Radiographs were developed using an automatic processor (Kodak X-OMAT 2000).

For the analysis of the radiographs, after their digitalization, a semiquantitative scale was applied to each gastrointestinal region (the stomach, small intestine, caecum, and the colorectal region) of each rat, and at each time-point, obtaining scores between 0 and 12, which were represented in the corresponding motility curves [27]. In addition, the alterations in the size and density of stomach, caecum, and fecal pellets were analyzed with the aid of an image analysis system (Image J 1.38 for Windows, National Institute of Health, USA, free software: https://rsb.info.nih.gov/ij/).

##### In Vivo Evaluation of Colonic Bead Expulsion 

Colonic bead expulsion test was performed, as described previously [29]. Briefly, on the day of the experiment, a pre-warmed (37 °C) glass bead (diameter: 8 mm) with a fire polished end was inserted 3 cm into the distal colon, using a silicone pusher [30]. Before insertion, beads were covered with Vaseline, to avoid tissue damage. After bead insertion, rats were separated into transparent, individual cages and the time to bead expulsion was measured [31]. Animals were monitored for a maximum of 4 h, unless the bead expulsion occurred sooner. Bead insertion was performed under sedation, with a Sedator^®^ (medetomidine hydrochloride, an alpha-2 adrenergic agonist, 1 mL/kg, 1 mg/mL, ip); immediately after bead insertion, a Revertor^®^ (atipamezole hydrochloride, an alpha-2 adrenergic antagonist, 0.66 mL/kg, 5 mg/mL, ip) was used to revert the sedation [32].

##### Extraction of Organs at Sacrifice

At the end of the study, animals were guillotined under anesthesia with sodium pentobarbital (2 mL/kg), the gastrointestinal package was extracted for evaluation of the length of the small intestine and colon, and samples were obtained for further studies.

### 2.8. Statistical Analysis

GraphPad Prism program version 5.01 (GraphPad Software Inc., San Diego, CA, USA) was used for statistical analyses. Results are shown as means ± standard deviation (SD) or ± standard error of mean (SEM). Differences between groups were analyzed using Student’s T test for unpaired data. A one- or two-way analysis of variance (ANOVA) followed by Bonferroni (gastrointestinal motility analysis) or Tukey’s (intracellular ROS analysis) test for mean comparisons was used to highlight the significant differences among samples. Differences were considered to be significant at *p* < 0.05 and highly significant at *p* < 0.01.

## 3. Results and Discussion

### 3.1. Coffee Silverskin Melanoidins Recovery

For recovery of the melanoidin-rich macromolecular fraction, five batches of 12 L of clarified crude coffee silverskin extract were submitted to ultrafiltration, with the equipment, and at the operating conditions already described in the Materials and Methods section. Concentration was carried out at 0.8 bar of transmembrane pressure, for an average mean time of 2 h. Even the initial extract had relatively low content of total dissolved (0.6–0.8 g/100 mL) and suspended solids (20–25 Nephelometric Turbidity Units (NTU)); the filtration flux declined quickly from 18 to 8 L/hm^2^. Addition of demineralized water during the diafiltration process did not improve filtration, maintaining the flux at levels between 8 and 2 L/hm^2^. This finding suggests that the gel layer built on the membrane surface was quite stable. Therefore, another 3 to 4 h were necessary to completely separate the low molecular weight species from the macromolecular fraction. 

Measurement of total soluble substances (TSS) of the permeate and concentrate streams at the beginning and the end of the concentration phase showed that the 10 kDa membrane retained from 50% to 70% of the extract soluble matter, which corresponded to the retained macromolecular fraction. Diafiltration of this retentate, with demineralized water and freeze-drying of the low molecular-weight-free macromolecular fraction, allowed the recovery of 25 g solid dry matter, per batch. The extraction yield of the MEL fraction was 3.2%. In this way, 126 g of purified melanoidin-rich fraction were obtained for the in vivo experimental study.

TSS increased progressively, until it reached 2 °Brix, pH values were constant, ranging between 5.0–5.28, turbidity increased continuously, and the content of dissolved salts decreased progressively. No bibliographical references have been found regarding the pH values of CSE, although the pH values for coffee beverages ranged from 4.9–5.6 [33]. Hashimoto et al. described that acidity is a positive quality of coffee beverage and is attributed to CGA, citric, tartaric, and malic acids. Compounds that contribute to a higher or lower pH and acidity are classified as dissociable and non-dissociable ionic species; within the dissociable ionic compounds are CGA, citric acid, quinic acid, and trigonelline, whereas the non-dissociable ionic compounds include caffeine and arabinogalactan [34]. 

The MEL fraction was analyzed microbiologically to evaluate the safety of its use as a food ingredient. The permitted values for molds in teas are up to 10^4^ CFU/g of the product and, for mesophilic aerobic colonies (30 °C) the permitted values are up to 10^6^ CFU/g of the product; our results being below these established limits. With regard to the microbiological safety of coffee, only the maximum permitted level for ochratoxin A (OTA) is legislated. Previous studies carried out by our research group showed values of 4.3 × 10^3^ CFU/g for the total aerobic microorganisms and a content of yeasts and molds lower than 10^2^ CFU/g in CSE [35], and OTA was not detected [5]. Table 1 shows the microbiological analyses of MEL beverages, according to current legislation for tea and its derivatives, regulated by RD 1354/83, BOE 05/27/83, and BOE 14/07/84. 

**Figure 1:**
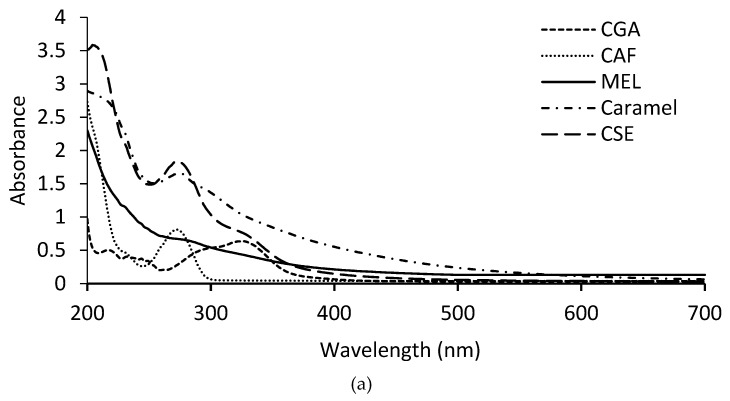

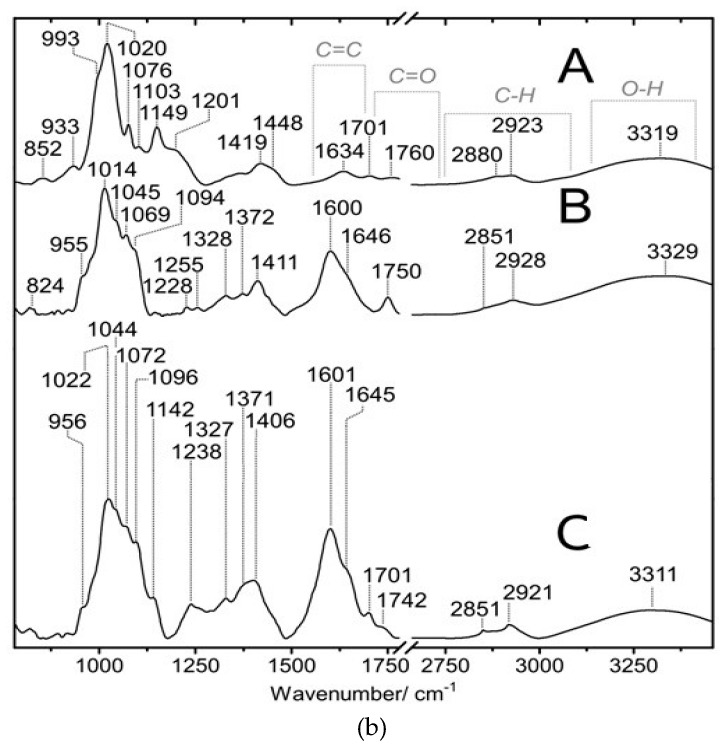
(**a**) UV-VIS spectra of caramel, melanoidins (MEL), coffee silverskin extract (CSE), caffeine (CAF), and chlorogenic acid (CGA). (**b**) IR spectra of caramel (A), coffee silverskin extract (B), and melanoidins (C). Relevant bands and vibrational modes are indicated.

Figure 1a shows the UV-Visible spectrum of the CAF, caramel, CGA, CSE, and MEL. CAF spectrum shows a maximum absorbance at 273 nm and the CGA spectrum shows a peak at 326 nm. In CSE, these two peaks are also observed. The maximum absorption at 273 nm might be due to the presence of proteins, CAF, and caffeic acid, and the maximum absorption at 326 nm might correspond to the presence of CGA [36]. Both CAF and CGA are low molecular weight nonpolar compounds [36]. Furthermore, it was observed that CSE absorbs at 400–450 nm, which might be due to the presence of melanoidins, previously described to absorb in this wavelength interval [10]. MEL fraction obtained after diafiltration showed absorption in the whole spectrum (270–450 nm), which might indicate the presence of melanoidins and traces of caffeine. Caramel (E-150d) has a similar spectrum to that observed in MEL. Melanoidins are soluble anionic polymeric compounds that are brown in color. These compounds have a molecular weight higher than 10 kDa, and absorb in the interval of 400 and 450 nm [10].

As an extended approach to standard analytical characterization, a spectral analysis in CSE and MEL was performed using vibrational spectroscopy as a non-invasive technique in the middle of the infrared region. This technique has been widely used for the molecular characterization and determination of green and roasted coffee bean compounds [37]. In addition, infrared spectroscopy also provides relevant information on the extraction of biocomposites [38]. Little is known about the implementation of infrared spectroscopy in the structural characterization of coffee by-products and the sustainable management of food, such as coffee silverskin. Figure 1b shows one of the first high-quality IR spectra of MEL (Figure 1B), compared with the IR spectrum of CSE (Figure 1C) and with the IR spectrum of the caramel standard (Figure 1A). Despite a similar brown coloration between the caramel and the MEL, a substantially different vibratory pattern was observed. In the caramel spectrum, a poor or pseudo-melanoidinic band was found, which suggests that all melanoidin extracts related to coffee show an equal mixture of reducing sugars and amino acids. In the CSE, a wide characteristic of C-O was observed around 1030 cm^−1^, but the maximum was displaced to 1022 cm^−1^, with local maximums of 1044, 1072, and 1096 cm^−1^. In addition, another prominent maximum was observed at 1601 cm^−1^, with one shoulder at 1645 cm^−1^ and two high-frequency peaks at 1699 and 1734 cm^−1^. The first peaks corresponded to C = C stretch characteristics, while the latter corresponded to stretch modes C = O. In addition, a broad peak was observed at 1406 cm^−1^, possibly to a flowering mode C-H_2_/CH_3_. In the high frequency region, little intense CH stretching peaks were detected at 2921 and 2851 cm^−1^ and a wide peak of OH stretching was seen at 3311 cm^−1^. After the extraction, no changes in the vibratory characteristics were observed, which suggests that the composition of the chemical prevailed. Both, CSE and MEL showed melanoidin characteristics. In addition, two different samples of each extraction showed almost identical characteristics. The structure of the coffee parchment, a by-product of the coffee bean, was analyzed by infrared spectroscopy and it was concluded that the components presented were lignin and cellulose. The bands at 1658 and 1602 cm^−1^ were assigned to lignin. In the high frequency region, the peaks at 2896 and 2712, were assigned to cellulose [5]. Taking into account that the dietary fiber of coffee has been described to be composed mainly of cellulose, hemicellulose, pectic substances, and lignin [12], and presents values close to those found for the parchment, it could be suggested that lignin is present in its structure.

**Figure 2:**
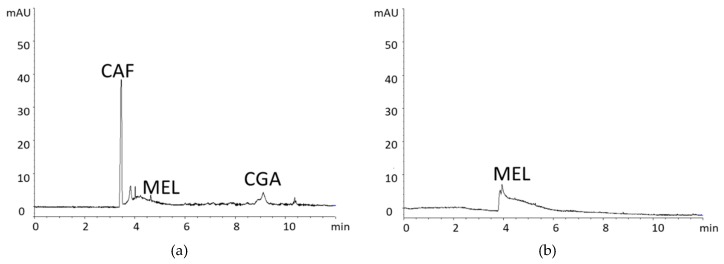
E-gram recorded at 280 nm showing the identified compounds from coffee silverskin (4 mg/mL) (**a**) and melanoidins (4 mg/mL) (**b**). Peak identification: CAF—caffeine; CGA—chlorogenic acid; and MEL—melanoidins.

CAF and CGA were determined by CZE. Identified compounds are shown in Figure 2. The e-gram of CSE showed two peaks, with a spectrum matching CAF and CGA, migrating at 3.4 min and 9.1 min, respectively. For comparative purposes, the identified compounds of the CSE were quantified by CZE (Table 2). CAF and CGA content in coffee CSE were 34.87 ± 1.85 mg/g extract and 11.50 ± 0.98 mg/g extract, respectively. In addition, a broad band was observed after 4 to 8 min, similar to that of coffee drinks and representing the colored macromolecules formed in the Maillard reaction, that is, melanoidins, as is described in the literature [39]. These spectral characteristics of the melanoidins confirmed its presence in CSE. Figure 2b shows the MEL isolate and, as expected, the same broad band is observed after 4 min, as in CSE, its maximum being found at 4.8 min.

Table 2 shows the chemical composition of CSE and MEL and their antioxidant capacity. Results indicate that melanoidins, proteins, CGA, and CAF are found in CSE. The amount of melanoidins present in CSE corresponds to 7.8 g/100 g, similar to that previously described [40]. However, no bibliographical references have been found regarding the isolation and characterization of coffee silverskin melanoidins. The caffeine content corresponds to that previously described by Mesías et al. [41]. The European Food Safety Authority (EFSA) has established a level of safety for daily caffeine consumption of 400 mg, for the general population, and 200 mg for lactating women [42]. Previous studies have indicated that the caffeine content in coffee byproducts are not worrisome [43]. The results obtained for CGA are lower than those found in other studies [40]. The antioxidant capacity of CSE is partially due to the presence of compounds generated during the Maillard reaction, melanoidins [40]. 

MEL presented low levels of CGA, CAF, and proteins, and a high melanoidin content (Table 2). The low values of protein (1.7 g/100 g) suggest that MEL is mainly composed of polysaccharides. In previous investigations, the high molecular weight fraction compounds were isolated from the coffee beverage (Arabica variety) and the main compounds found were polysaccharides (37 g/100 g), followed by proteins (12 g/100 g) [44]. 

The main compound present in the MEL fraction obtained in this study, was TDF (75.1%). Recommendations regarding the consumption of TDF vary, depending on the regulation. For instance, the World Health Organization (WHO) recommends a daily intake of 27 to 40 g of TDF, the Food and Drug Administration (FDA) recommends a consumption of 25 g of TDF per day, while the American Dietetic Association (ADA) recommends consuming between 20 and 30 g/day. Among the TDF content, IDF and SDF were 56.6% and 18.5%, respectively.

According to the literature, the CSE has a TDF value of 62.4 g/100 g, the IDF content being higher (53.7 g/100 g) than the SDF content (8.8 g/100 g) [45]. No studies in the literature were found to have looked into the chemical characterization of the MEL fraction from the roasting by-product of coffee processing. Low molecular weight compounds, such as CGA, adhere to the structure of melanoidins during roasting, leading to an antioxidant dietary fiber. Coffee silverskin fiber has been previously classified as an antioxidant dietary fiber [46,47]. SDF and IDF have different physiological functions, largely depending on the viscosity of fibers. In the large intestine, they promote a regulatory effect: IDF (found in cellulose, hemicellulose, lignin, and resistant starch) mechanically excites the intestinal mucosa, by stimulating it and leading to the secretion of water; and SDF (found in inulin, pectins, gums, and fructooligosaccharides) forms a gel with a high water retention capacity. To exert its regulatory effect, dietary laxative fibers must resist fermentation, and remain relatively intact and present throughout the large intestine, consequently, increasing the water content in feces [48]. In this study, an isolate enriched extract in the Maillardized dietary fiber was obtained, from the coffee silverskin. Taking into account the physiological properties of SDF and IDF in gastrointestinal health, it is of great interest to evaluate the bioactivity of this sustainable source of fiber in vitro and in vivo.

### 3.2. Bioactivity of Coffee Silverskin Melanoidins

#### 3.2.1. Antioxidant Capacity

The in vitro antioxidant capacity of CSE and MEL, determined by ABTS and ORAC, is shown in Table 2. Antioxidant capacity was significantly higher (*p* < 0.05) in CSE, compared to MEL. This trend was observed for both methods. Results regarding the antioxidant capacity of CSE agreed with those described in the literature. The antioxidant capacity of CSE might be due to the phenolic compounds present in the raw material and due to the presence of melanoidins formed during roasting [12]. It has been suggested that these low molecular weight phenolic compounds, such as CGA, are covalently bound to the carbohydrate backbone, forming a fiber–antioxidant complex [49]. Since the MEL fraction obtained in this study was diafiltrated, non-bound low molecular weight compounds were eliminated and, therefore, it was expected to have a lower antioxidant capacity. Even so, results indicated that MEL has antioxidant properties and contributes about 35%, to the total antioxidant capacity of CSE. No bibliographical references have been found regarding the total antioxidant capacity of MEL from the coffee silverskin.

**Figure 3:**
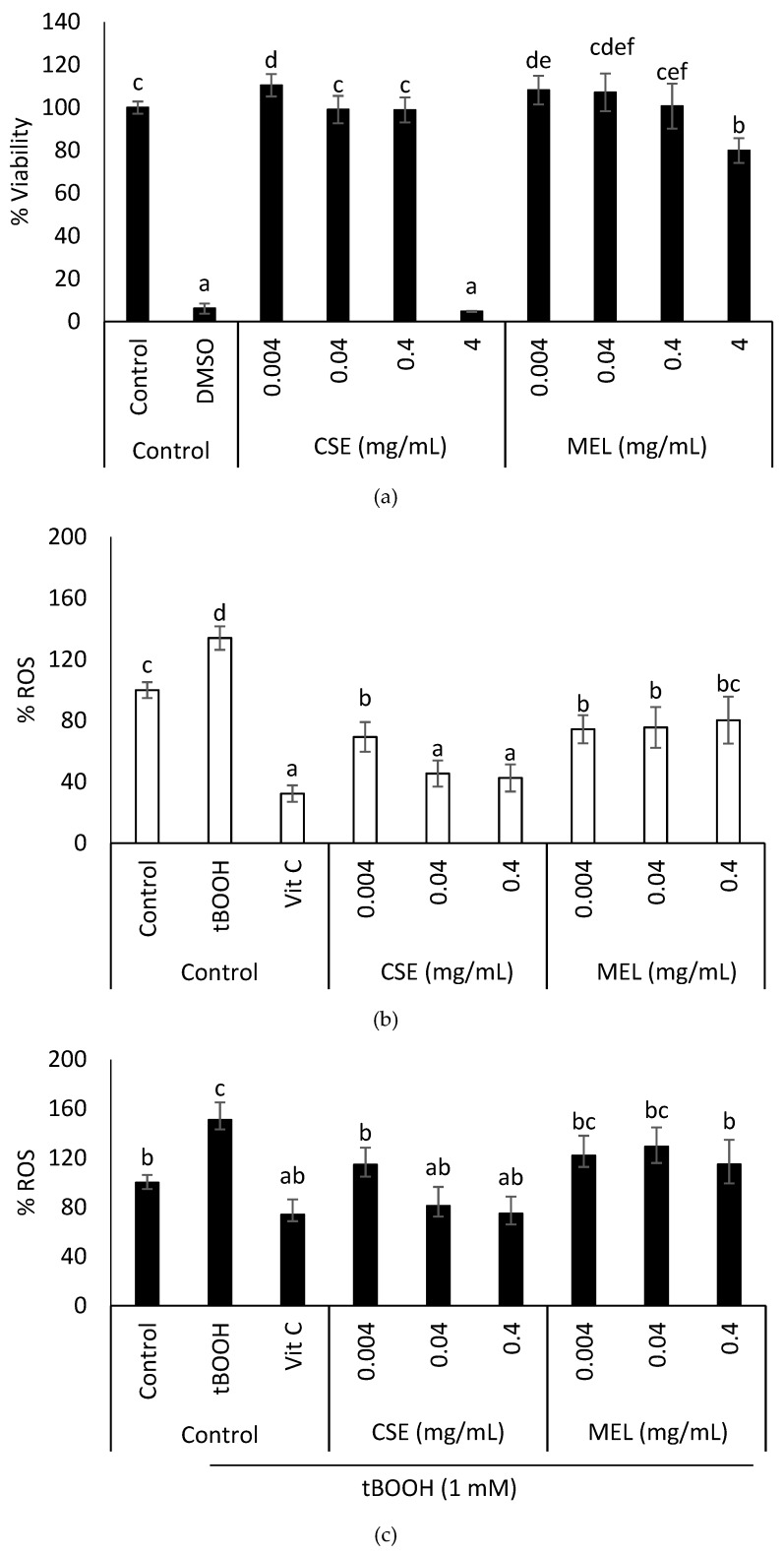
Effect of CSE and MEL on IEC-6 (**a**) cell viability, (**b**) physiological ROS, and (**c**) induced intracellular ROS. Cells were cultured with different concentrations of CSE or MEL, for 24 h. Then, the MTT assay was performed to determine cell viability and ROS were measured using the DCFH-DA probe. Control—untreated cells; death control—DMSO (50%); oxidation control—tBOOH (1 mM); antioxidant control—vitamin C (10 µg/mL). Data are shown as the mean ± SEM of the three independent experiments. Different letters above columns indicate significant differences among treatments (Tukey test, *p* ≤ 0.05).

Prior to the evaluation of the effect of MEL on the intracellular ROS formation, cell viability assays were performed to select non-cytotoxic concentrations of extracts. Figure 3a shows the viability of healthy rat intestinal cells (IEC-6), after a 24 h treatment with CSE and MEL, at different concentrations. The highest concentration of CSE and MEL (4 mg/mL) significantly reduced (*p* < 0.05) cell viability, when compared to the non-treated control cells. Therefore, lower concentrations of samples (0.004, 0.04, and 0.4 mg/mL) that resulted in non-cytotoxic intestinal cells, were selected for the study of their effect on the redox status of cells.

Figure 3b shows the effect of CSE and MEL on the physiological ROS of IEC-6 cells, after 24 h of incubation. Both extracts significantly reduced (*p* < 0.05) the physiological production of ROS, compared to the control (untreated cells). Concentrations of 0.04 and 0.4 mg/mL of CSE had a similar antioxidant effect (*p* > 0.05) as vitamin C (10 µg/mL) used as an antioxidant control.

The effect of CSE and MEL on induced intracellular ROS of IEC-6 cells is shown in Figure 3c. Oxidation was induced by tBOOH 1 mM, which significantly increased the production of intracellular ROS (*p* < 0.05). As expected, vitamin C significantly reduced the formation of induced ROS (*p* < 0.05). When intestinal cells were pre-treated with different concentrations of CSE (0.004, 0.04, and 0.4 mg/mL), a significant reduction of induced ROS was observed (*p* < 0.05). The reduction of ROS levels by CSE has also been studied in other cell lines, such as liver and skin cells [5,50]. Our results agreed with those described for these other cell lines. In addition, induced levels of ROS were also decreased when cells were pre-treated with MEL at 0.4 mg/mL. Results seem to indicate that the low molecular weight compounds present in the CSE were removed during the purification process, such as CGA, that might have contributed to the antioxidant effect observed on the intestinal cells. To date, no other study has been found in the literature that has looked into the effect of MEL from coffee silverskin on induced intracellular ROS. Thus, the MEL fraction obtained from the coffee silverskin in this study, shows in vitro antioxidant capacity and is effective against induced ROS.

#### 3.2.2. Toxicity and Fiber Effect

In this study, MEL was administered in the drinking water in a non-invasive manner, as previously described. Previous studies have also administered samples in this way, for instance, freeze-dried instant coffee was administered to animals in a solution at 1.2 g/kg of body weight [51]. 

The mean weight of the animals at the beginning of the study was 186 ± 6.27 g (control group) and 174 ± 5.99 g (MEL group). Throughout four weeks of the in vivo study, there was a gradual increase in weight in both groups of animals, without showing statistically significant differences in this parameter, due to the exposure of the animals to MEL in the drinking water (Table S1 shows final weight: Control: 321.8 ± 8.5; MEL: 318.6 ± 16.4; *p* > 0.05). With regards to food intake, no statistically significant differences were observed, either (*p* > 0.05). Food intake of the control group was 24.24 ± 0.95 g/day/rat and that of the group with MEL was 22.04 ± 0.96 g/day/rat. The group treated with the MEL beverage presented an intake of 37.06 ± 2.33 mL/day/rat and the control group showed an intake level 34.03 ± 1.14 mL/day/rat. A tendency of a higher liquid consumption in the group treated with MEL beverage, compared to the control group was observed, but the difference was not statistically significant (*p* > 0.05). To date, there are no in vivo studies with coffee silverskin melanoidins. However, the effect of coffee beverage consumption in vivo, has been addressed. Animals treated with the coffee beverage for four weeks, showed a lower body weight and food intake, when compared to the control animals. This might be the result of a decreased food intake, due to a loss of appetite in the animals with coffee beverage, since the consumption of caffeine, one of the major biologically active components of coffee, reduces energy intake by 22% [51]. According to the literature on studies carried out with fructans with an inulin content of ≥90%, rats treated with a high fiber diet, three times per week for eight weeks, showed a significantly lower consumption of food, compared to the control group [52,53]. Castillo Andrade et al. described that this effect might be due to the Glucagon-like peptide type 1 (GLP-1) production that increases satiety and reduces energy intake. Authors also observed that the treated group showed a higher water intake, which might be associated with a high fiber consumption [54]. Our results seemed to indicate that the slight decrease in food consumption might be due to the presence of MEL, and its high fiber composition might explain the slight increase in drinking.

Throughout the study, no sign of clinical dehydration was found in any of the rats and the appearance of the perianal area, as well as that of the fecal pellets [26] were normal. Table S1 shows the weights of the different organs normalized to the weight of the corresponding rat, at sacrifice. Compared to the control animals, in those treated with MEL, there was a significant decrease in the weight of the small intestine, probably due to a concomitant decrease in its content (milking), which could be associated with an increased intestinal transit (see below). Even though most other organs showed no macroscopic modifications, the liver and kidneys presented slightly lower weight in MEL (although the difference was not statistically significant for the left kidney). No other study has been found in the literature that has looked into coffee silverskin melanoidins; however, studies on CSE, have found that CSE at 2 g/kg did not produce visible signs of toxicity, nor did they find differences between the weights of the different organs (*p* > 0.05) [5]. While liver and kidney weights were not measured in studies carried out in rats exposed chronically to a high fiber diet (fructans with an inulin content of ≥90%), the ability of hepatocytes to biosynthesize plasma proteins or renal tissues to remove waste substances, was not affected [52,54]. The lack of major differences in the weight and appearance of the different organs suggest that exposure to MEL beverage did not produce a meaningful toxicity.

**Figure 4:**
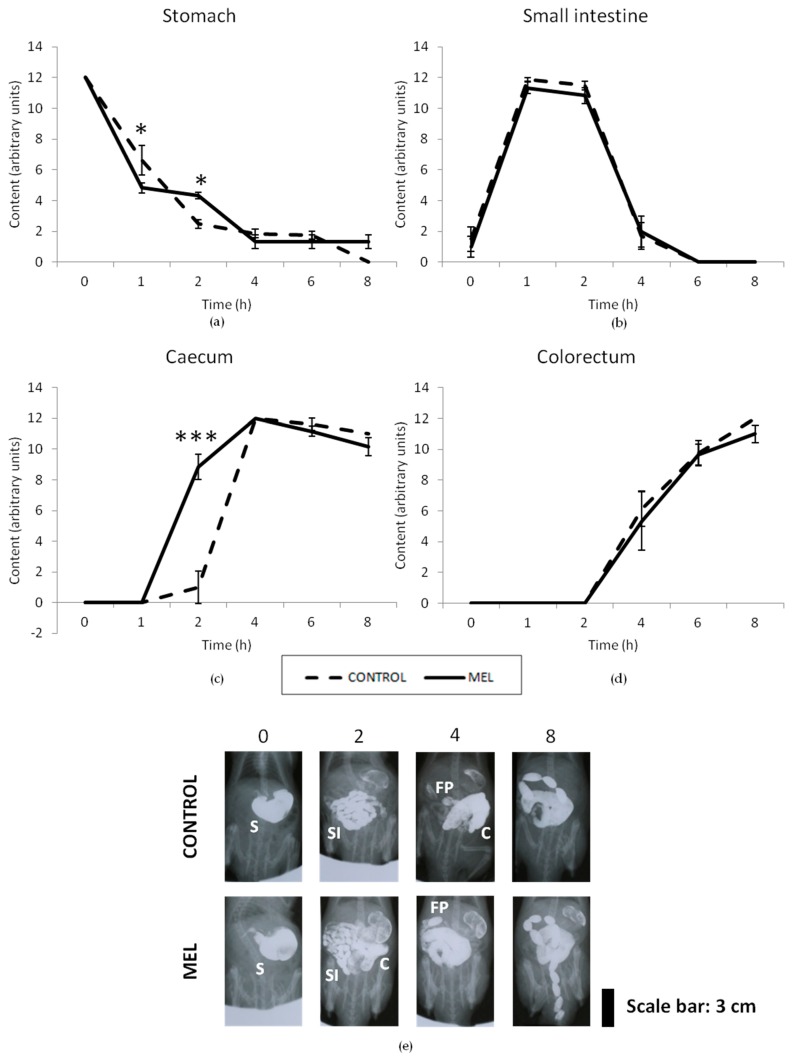
Radiological analysis of gastrointestinal motor function in rats—a semiquantitative analysis. A dose of barium sulfate (3 mL, 2 g/mL) was intragastrically administered at time 0, and X-rays were taken immediately and 1, 2, 4, 6, and 8 h, after administration. Motility curves for the stomach (**a**), small intestine (**b**), caecum (**c**), and colorectum (**d**) were obtained from the control rats (*n* = 8) and rats treated with MEL (4 g/L) (*n* = 6). Data represent the mean ± SEM. * *p* < 0.05, *** *p* < 0.001 (Two-way ANOVA followed by Bonferroni post-hoc test). Representative X-rays (**e**) obtained from a control and a MEL-treated rat at 0, 2, 4, and 8 h, after administration of barium sulfate. S—stomach; SI—small intestine; C—caecum; FP—fecal pellets (within the colorectum).

**Figure 5:**
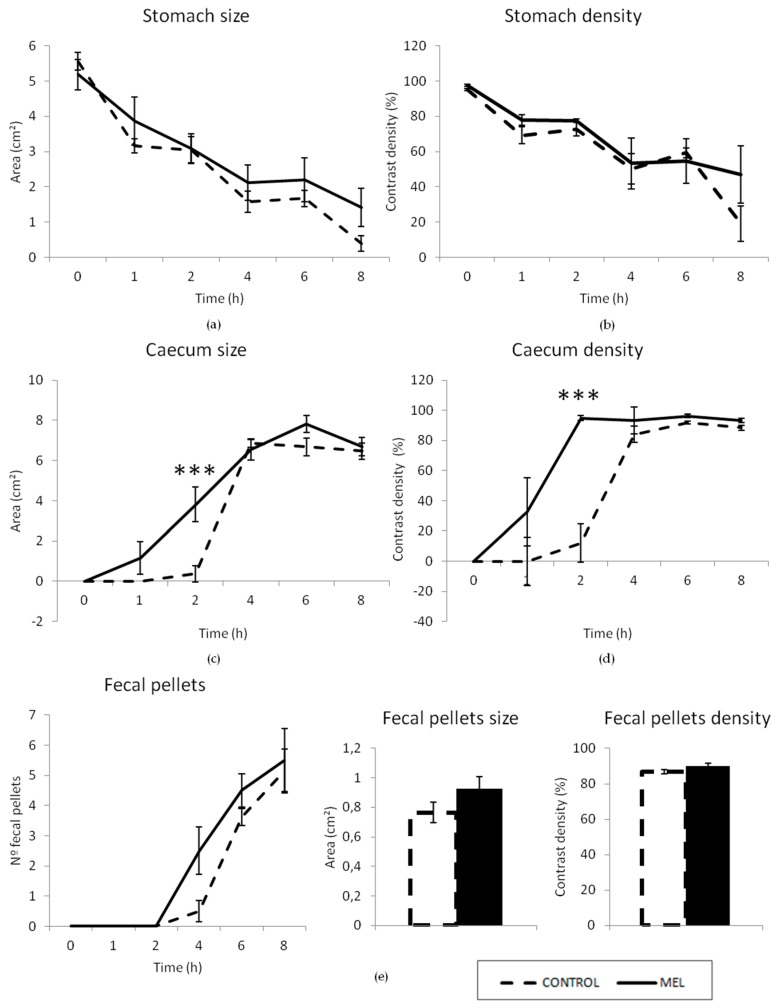
Radiological analysis of gastrointestinal motor function in rats—morphometric and densitometric study. A dose of barium sulfate (3 mL, 2 g/mL) was intragastrically administered at time 0, and X-rays were taken immediately and after 1, 2, 4, 6 and 8 h from time of administration. Morphometry and densitometry analyses of the stomach (**a**,**b**), caecum (**c**,**d**), and fecal pellets (**e**) are shown for the control rats (*n* = 8) and the rats treated with MEL (4 g/L) (*n* = 6). Size and density were evaluated using Image J (see text for details). Data represent the mean ± SEM. *** *p* < 0.001. (Line graphs: Two-way ANOVA followed by Bonferroni post-hoc test. Bar graphs: Student’s *t*-test).

The fiber effect of melanoidins was specifically studied using radiographic analyses and the bead expulsion test, after 4 weeks of exposure to MEL. Results of the radiographic study are shown in Figure 4 and Figure 5. Results of the semiquantitative analysis of the stomach, for the control group, showed a progressive emptying of barium, throughout the 8 hours of the radiographic session. Compared to control animals, the group treated with the MEL beverage showed statistically significant differences for time points 1 and 2 (*p* < 0.05), although, this difference was possibly negligible, from a clinical point-of-view (Figure 4a). The motility curves for the small intestine showed a filling phase (0–1 h), a plateau (1–2 h), and a progressive emptying phase (2–8 h); these were practically overlapped for both groups, throughout the radiographic session, without showing any statistically significant difference at any time-point (*p* > 0.05) (Figure 4b). Figure 4c shows the motility curve for the caecum. In the filling phase, significant differences were observed (*p* < 0.01), since it took four hours for the caecum of the control animals to be completely filled-up, whereas that of the animals treated with MEL beverage, was practically filled-up in only two hours. Thereafter, the caecum remained practically full, until the end of the study, in both groups, with no statistically significant differences between them. With regards to the colorectum, the control group began to form fecal pellets two hours after contrast and reached the maximum score at 8 hours of the radiographic session (Figure 4d). The curve for the colorectum of MEL group overlapped with that of control animals, without any statistically significant difference (*p* > 0.05).

The alterations in the size and density of stomach, caecum, and fecal pellets are shown in Figure 5. No significant differences were found for morphometry and densitometry in the stomach, between the control and the MEL groups (*p* > 0.05). Figure 5c,d show the curves for the caecum size and density, respectively. Significant differences were observed (*p* < 0.01) after 2 h. These results agreed with the semiquantitative results shown in the motility curves for the caecum (Figure 4c), as described above. The intense folding of the small intestine in the abdomen did not allow for differences to be seen in the semiquantitative study, but barium reached and filled the caecum much faster in the MEL group, compared to the control group. This was not due to a faster gastric emptying (Figure 4a, Figure 5a,b) or to a reduction in the length of the small intestine (Table S1); most likely, the propulsive activity of small intestinal semisolid contents was increased by MEL. Additionally, the number of fecal pellets found within the colorectum was slightly bigger in the group treated with the MEL beverage than in the control group, at 4–8 h (at 4 h, the Student’s *t*-test showed that this difference was actually significant) (Figure 5e). This suggests that pellet formation was accelerated by MEL (differences might be highlighted by including additional time-points in the radiographic session, i.e., after 3 h). Although no significant differences in the density of fecal pellets were found, they tended to be bigger in the MEL group than in the control group (*p* = 0.1610, Student’s *t*-test; Figure 5e). These increased number and size of fecal pellets might be a result of the higher fiber intake in the MEL group, making them slightly more effective to mechanically stimulate the colon.

Previous studies have found that animals treated with fructans showed a higher fecal production and a softer consistency, compared to the control group [54]. Increased fecal excretion has been related to fiber consumption and its stimulating effect on the absorption of water and electrolytes from the lumen of the colon, which leads to a greater fecal viscosity and a general decrease in intestinal transit time [55]. In previous studies, the effect of coffee grounds of the Robusta variety on gastrointestinal motor function was studied. It was administered by gavage, at a dose of 1 g/kg, once a day from Monday to Friday, for four consecutive weeks. Compared to the vehicle, coffee grounds increased transit, after the first but not after the 14th and 28th administrations, suggesting the development of tolerance to the laxative effect [56]. In the present study, transit was increased, even after 28 days of MEL exposure in drinking water. Further studies are warranted to determine the effects of MEL at different exposure times.

There were no statistically significant (*p* > 0.05) differences in the lengths of the small intestine and the colon, between the control group and the MEL group (Table S1). Previous studies with fructans of *Agave salmiana* in the Wistar rats, for 35 days, observed that the size and length of the caecum and the colon were bigger, with respect to the animals without treatment. This was considered a positive effect because it indicated a larger area available for the absorption of nutrients and the increase of the beneficial intestinal microbiota, due to the consumption of fructan, and concluded that this fermentation began in the caecum [54]. More research is needed to determine if a longer exposure to MEL might induce similar results. 

Finally, the bead expulsion times were 470 ± 141 s for the control group and 502 ± 142 s for the MEL group, with no statistically significant differences between both groups (*p* > 0.05). In this test, the pellet was inserted only 3 cm into the colorectum, from the anus, in a region where, under normal conditions, the fecal pellets are already formed and, possibly, the effect of the MEL beverage occurs in regions closer to the caecum, where the aqueous content of the feces can still be greatly altered. The fact that MEL beverage did not modify this parameter also indicated that the motor agents (intrinsic and extrinsic innervation, smooth muscle and interstitial cells of Cajal or pacemakers, [57]) involved in the colonic propulsion, at this level, are intact and respond similarly to the same mechanical stimulus (8 mm diameter-bead). This might not be the same for the small intestine, whose propulsive activity was increased, as shown above, and more studies are needed to identify the specific mechanism involved. Whatever the case may be, no other study has been found on coffee, coffee silverskin, or coffee silverskin melanoidins, for this parameter.

## 4. Conclusions

This study provided novel information on the characterization of coffee silverskin melanoidins. Microbiological quality and health status of the treated animals support the food safety level of high molecular weight compounds, including melanoidins. The conditions for the isolation process were effective in obtaining a high molecular weight fraction (>10 kDa). The results of chemical, structural and functional characterization in vitro, showed the extraction of melanodins with antioxidant properties, which might be defined as the “Maillardized antioxidant dietary fiber”. In vivo pilot study results of intestinal motility confirmed an acceleration of the intestinal transit in animals treated with MEL, even after 28 days of exposure. Coffee silverskin melanoidins have the potential to be used as a functional food ingredient.

## Figures and Tables

**Table 1:** Microbiological analyses of the melanoidin beverage (MEL) prior to its administration to rats.

Microbiological Analyses	MEL
Molds (CFU/g)	<10^2^
Yeasts (CFU/g)	4.8 × 10^4^
Total aerobic microorganisms (CFU/g)	4.8 × 10^4^
Endospores 30 °C (CFU/g)	<10
Viable aerobic microorganisms at 30 °C (CFU/g)	9.4 × 10^3^

**Table 2:** Chemical composition and in vitro bioactivity of coffee silverskin extract (CSE) and melanoidins (MEL).

	CSE	MEL
Measure (% *w*/*w*) *		
Caffeine	3.5 ± 0.2 ^a^	0.1 ± 0.0 ^b^
Chlorogenic acid	1.2 ± 0.1 ^a^	0.2 ± 0.0 ^b^
Melanoidins	7.8 ± 0.2 ^a^	14.6 ± 0.8 ^b^
Protein	2.2 ± 0.0 ^a^	1.7 ± 0.0 ^a^
Dietary fiber	N.D.	75.1 ± 4.8
**Total Antioxidant Capacity (µmol Trolox/mg Sample)**		
ABTS	4.0 ± 1.2 ^a^	1.8 ± 0.1 ^b^
ORAC	4.4 ± 0.2 ^a^	1.1 ± 0.0 ^b^

* Weight per freeze-dried sample. N.D.: not determined. Data represent mean ± SD of three independent experiments. Different letters represent statistically significant differences (Student’s *t*-test, *p* < 0.05).

## Supplementary Materials

The following are available online at http://www.mdpi.com/2304-8158/8/2/68/s1. Table S1: Effect of melanoidin consumption on body weight, normalized rat organs weight (g/kg rat), and length of the small intestine and colorectum (cm) of the control (*n* = 8) and the treated rats (*n* = 6). Values represent the mean ± SEM. Asterisk denotes significant difference compared to the control (Student’s *t*-test, * *p* < 0.05).
