# Supplementary material for: An Assessment of the Bioactivity of Coffee Silverskin Melanoidins
Source: Foods. 2019 Feb 12;8(2):68. doi: 10.3390/foods8020068 (PMC6406266; doi:10.3390/foods8020068)
Supplement: Supplementary file 1 [file foods-08-00068-s001.pdf]

foods

Table S1. Effect melanoidin consumption on body weight, normalized rat organs weight (g/kg rat) and length of the small intestine and colorectum (cm) of control ($n = 8$) and treated rats ($n = 6$). Values represent the mean ± SEM. Asterisk denotes significant difference compared to control (T-student, * $p < 0.05$).

	Control	MEL
Body weight (g)	321.8 ± 8.5	318.6 ± 16.4
Stomach (g/kg)	12.1 ± 1.0	10.1 ± 1.0
Small intestine (g/kg)	33.2 ± 1.0	29.4 ± 1.0 *
Milking[1] (g/kg)	8.4 ± 0.4	5.6 ± 0.6 *
Caecum (g/kg)	17.6 ± 0.8	16.4 ± 1.9
Colon (g/kg)	15.0 ± 1.2	13.6 ± 3.3
Salivary glands (g/kg)	2.4 ± 0.1	2.2 ± 0.2
Left kidney (g/kg)	3.3 ± 0.08	3.1 ± 0.04
Right kidney (g/kg)	3.2 ± 0.03	3.05 ± 0.06 *
Liver (g/kg)	38.8 ± 1.1	34.1 ± 1.0 *
Epididymal fat (g/kg)	21.9 ± 2.3	20.2 ± 1.7
Pancreas (g/kg)	4.0 ± 0.3	4.3 ± 0.4
Small intestine (cm)	58.7 ± 1.7	58.3 ± 0.9
Colorectum (cm)	14.5 ± 0.6	14.8 ± 1.4

[1] Milking: content of the small intestine.
